# NMR Identification and Quantification of Galactinol in Green *Coffea arabica*: Implications for Traceability and Adulteration

**DOI:** 10.1111/1750-3841.71238

**Published:** 2026-06-24

**Authors:** Elisabetta De Angelis, Simone Fabbian, Elisabetta Schievano, Elena Guercia, Arianna Fornasari, Elena Piva, Michele Pozzebon, Luciano Navarini

**Affiliations:** ^1^ Aromalab illycaffè spa Area Science Park Trieste Italy; ^2^ Department of Chemical Sciences University of Padova Padova Italy; ^3^ Department of Medical and Surgical Sciences University of Bologna Bologna Italy; ^4^ DTO Labs Spinea Italy; ^5^ illycaffè spa Trieste Italy

**Keywords:** ciceritol, *Coffea arabica* L., galactinol, HPLC–RID, NMR

## Abstract

Among coffee low‐molecular‐weight carbohydrates, galactinol plays an important role in coffee physiology and quality, having received attention also for its potential use as a marker of geographical origin. Nevertheless, data on the occurrence of this sugar in green coffee beans remain limited. In this study, galactinol was isolated for the first time from green *Coffea arabica* beans and unambiguously identified by high‑field NMR spectroscopy. A quantitative HPLC–RID screening of 100 green Arabica coffee samples from 16 geographical origins showed that galactinol was detected in 31% of the samples, with consistent occurrence in Ethiopian coffees but sporadic or absent detection in several other origins. Notably, the chromatographic analysis revealed a previously unreported co‐elution of galactinol with ciceritol, a chickpea‐derived cyclitol of relevance for coffee adulteration detection. These results highlight both the potential and the limitations of galactinol as a geographical marker and stress the importance of combining chromatographic and structure‑specific spectroscopic approaches to ensure reliable interpretation in coffee traceability and authenticity studies.

## 1 Introduction

Carbohydrates are the major constituents of green *Coffea arabica* L. (Arabica) beans, accounting for 59%–61% of their dry weight (DW). Among them, low‐molecular‐weight carbohydrates have been particularly studied for their role as aroma precursors. Sucrose (7%–8% DW) is by far the most representative member of this group, accounting for more than 90% of total low‐molecular‐weight carbohydrates, followed by small amounts of raffinose family oligosaccharides (RFOs), such as stachyose (0.04%–0.2% DW) and raffinose (0.02%–0.06% DW) (Knopp et al. 2006; Rogers et al. 1999).

Among coffee low‐molecular‐weight carbohydrates, the galactosyl cyclitol known as galactinol (1‐*O*‐α‐d‐galactopyranosyl‐l‐*myo*‐inositol) has attracted considerable attention owing to its relevance across multiple aspects of coffee science. Galactinol has been associated with postharvest processing methods and cup quality attributes in specialty coffee (Amalia et al. 2021; Amalia et al. 2023) and plays a key role in coffee seed physiology as a galactosyl donor in the biosynthesis of RFOs, potentially contributing to seed desiccation tolerance and longevity (Chabrillange et al. 2000; Gojło et al. 2015). From an analytical perspective, galactinol has also been suggested as a potential marker of geographical origin in green *C. arabica* L. An untargeted metabolomics analysis found that galactinol is correlated with coffee Arabica beans from Santo Antônio do Amparo, a specific region in Brazil, and may be a potential metabolite marker of three different Bourbon genotype lines when compared with the Brazilian cultivar Mundo Novo (Da Silva Taveira et al. 2014). Moreover, gas chromatography/mass spectrometry (GC/MS) metabolic profiling indicated that galactinol correlates with the geographical origin of coffee beans from different Indonesian regions (Putri et al. 2019).

Notably, despite the relevance of galactinol from coffee seed physiology to coffee cup quality, it has not yet been isolated and quantified from green Arabica coffee beans through targeted approaches. In addition, reliable quantitative data on a sufficiently wide pool of coffee samples to investigate the potential role of this sugar as a geographical marker have not been reported so far.

To address these research gaps, we employed an integrated chromatographic–NMR approach to structurally identify, isolate, and quantify galactinol from green Arabica coffee beans and to provide preliminary quantitative data on its occurrence across a selection of 100 commercial samples from 16 different geographical origins. Chromatographic analysis was performed using quantitative HPLC/RID screening under analytical conditions normally used to profile soluble sugars in RFO‐rich food matrices (Sánchez‐Mata et al. 1998; 2002). Structural validation was achieved by leveraging CSSF‐TOCSY (Chemical Shift Selective Filtered Total Correlation Spectroscopy) NMR experiments (Scettri and Schievano 2022; Schievano et al. 2017, 2020; Robinson et al. 2004). To evaluate the specificity of galactinol as a potential geographical and authenticity marker of green Arabica coffee, its presence was also evaluated in chickpeas (*Cicer arietinum*), a well‐known coffee adulterant (Sezer et al. 2018), by subjecting a soluble extract to the same HPLC–RID and NMR analyses. Although the comparison of chromatographic profiles initially suggested the presence of galactinol also in chickpea, subsequent NMR analysis demonstrated that the corresponding chromatographic signal was instead attributable to ciceritol, a naturally occurring d‐pinitol digalactoside found in legume seeds but not reported in coffee. This previously unreported co‐elution of galactinol and ciceritol prompted the development of an optimized HPLC–RID method capable of resolving the analytical interference, thereby enabling the selective determination of galactinol in green Arabica coffee beans and minimizing potential interferences from noncoffee cyclitol galactosides arising from adulteration practices (Sezer et al. 2018).

The main objective of this work was to advance current knowledge on galactinol in green *C. arabica* beans through its first unambiguous identification, isolation, and systematic quantitative screening across a relatively large and geographically diverse sample set, with particular emphasis on its potential as a geographical marker for traceability and authenticity assessment. Our findings revealed that, with the exception of Ethiopian varieties, galactinol levels did not consistently correlate with geographical origin, suggesting that its role as a traceability marker should be considered with caution. Importantly, the co‐elution of galactinol with ciceritol, observed in this study for the first time to the best of our knowledge, highlights the need to integrate high‐resolution structural techniques such as NMR spectroscopy into coffee authenticity studies. Such an approach would minimize the risk of misinterpretation associated with relying solely on chromatographic sugar profiles.

## 2 Materials and Methods

### 2.1 Materials

Ethanol 96% extra pure, methanol for HPLC ≥99.9%, silica gel 60 (70–230 mesh), sea sand pro analysis, and raffinose (pentahydrate) were purchased from Merck (Rome, Italy). Ammonium hydroxide solution max 33% NH_3_ ultrapure, 1‐butanol, naphthoresorcinol, galactose, sulfuric acid 99%, *myo*‐inositol and sucrose were purchased from Sigma–Aldrich (Saint Louis, MO, USA). Acetone for analysis (ACS reagent), ethanol absolute anhydrous, and acetonitrile for LC/MS were purchased from Carlo Erba reagents (Milan, Italy). Stachyose hydrate technical was purchased from Acros Organics (Thermo Fischer Scientific, Waltham, MA, USA). Ultrapure water was obtained using a Milli‐Q Academic Merck apparatus (Merck Millipore, Molsheim, France). Deuterated water (D_2_O, 99.90%) was supplied by Eurisotop.

### 2.2 Samples

A total of 100 green coffee (*Co. arabica* L.) commercial lot samples from 16 different geographical origin were used: Brazil (12), Rwanda (12), Vietnam (5), Costa Rica (7), Nicaragua (11), Mexico (4), Honduras (8), El Salvador (3), Ethiopia (13), Colombia (5), India (6), Guatemala (8), Congo (1), Perú (1), the Dominican Republic (1), and Uganda (3). The samples supplied by illycaffè S.p.A. Quality Control Department (Trieste, Italy) were wet processed with zero primary and secondary defects and selected based on standard procedures of sorting and visual aspect, moisture content, screen size, and cup quality. Green coffee beans were ground in a ball mill (Retsch MM400; grinding jar: stainless steel, 50 mL, screw top design; grinding ball: Ø 25 mm, stainless steel; frequency 30.0 Hz; time 20 s) to fine powder. Then, 1 g was mixed with 25 mL of boiling Milli‐Q water and put in an orbital shaker (Heidolph Unimax 1010, Schwabach, DE) for 90 min at 24°C. Samples were transferred to 50‐mL tubes and centrifuged (Allegra 64R Centrifuge, Beckman Coulter, Indianapolis, IN, USA) for 8 min for 7000 rpm. After centrifugation, an aliquot of the supernatant was passed through a 0.45‐µm nylon membrane filter (Phenomenex, Torrance, CA, USA) to provide aqueous solutions that were transferred in 2‐mL vial ready for analysis. All samples were prepared and analyzed in triplicate.

Chickpea (*Ciceri arietinum* L.) dried seeds used to ascertain the presence of galactinol and to isolate ciceritol to be used as a chromatographic standard were purchased in a local market.

### 2.3 HPLC–RID

Analyses were carried out by using an Agilent 1260 series HPLC system equipped with a refraction index detector (RID) (Agilent Technologies, Santa Clara, CA, USA). For sugar separation, the method according to Quemener and Brillouet (1983), with some modifications, was used. In particular, the following conditions were used: a ZORBAX‐Carbohydrate analytical column (5 µm, 4.6 × 250 mm; Agilent Technologies) kept at 30°C with acetonitrile–water (60:40, w/w) as the mobile phase at a flow rate of 1.0 mL/min and injection volume of 5 µL. Sugars were identified using individual standard solutions. For the sake of brevity, we named this method “Method A.”

Additional analyses (“Method B”) were performed using an Agilent 1100 HPLC system equipped with an Agilent 1260 RID. The column was an Agilent Hi‐Plex Ca (300 × 7.7 mm). Ultrapure water was used as the mobile phase. The RID operated with positive polarity and an optical temperature of 55°C. The column oven temperature was set at 80°C. The flow rate was set to 0.4 mL/min, and the injection volume was 10 µL. The run time for the analysis was 30 min. Data analysis was performed using Agilent ChemStation software. The HPLC–RID system was allowed to equilibrate overnight under the analysis conditions to ensure a stable RID signal.

#### 2.3.1 Quantification of Galactinol by Method A

For quantification, galactinol from TCI (Tokyo Chemical Industry, Co., Ltd.) as a standard was used. A standard stock solution was prepared by dissolving galactinol in Milli‐Q water (0.64 g/L). The linearity of the method was evaluated by analyzing six standard solutions with concentrations ranging from 0.64 to 0.013 g/L in Milli‐Q water. A diluted solution was freshly prepared and analyzed with samples to confirm the RT of the galactinol peak.

Limits of detection and quantification (LOD and LOQ) values were calculated using the residual standard deviation (*S_y_*_/_ *_x_*) and the slope (*b*) of the calibration curve (*R* ^2^ = 0.99997) as LOD = 3(*S_y_*_/_ *_x_*)/*b* and LOQ = 10(*S_y_*_/_ *_x_*)/*b*. The LOD and LOQ values determined for galactinol were 0.005 and 0.015 g/L, respectively.

The repeatability was expressed as relative standard deviation (RSD% = SD/mean × 100). The RSD of a calibration standard (*n* = 10) for intraday precision was 4.1% while the RSD of a representative coffee sample (*n* = 10) was 29%.

### 2.4 Mass Spectrometry (ESI–MS)

Ciceritol was dissolved in Milli‐Q water: acetonitrile 50:50 with 0.1% (v/v) formic acid. MS analysis was carried out in positive ion mode by direct infusion electrospray ionization (ESI) on an Agilent 6550 iFunnel Q‐TOF mass spectrometer. Data were finally processed by using Mass Hunter software.

### 2.5 NMR Spectral Acquisition and Signal Processing

One‐ and two‐dimensional NMR spectra, in deuterated water, were recorded on a Bruker Avance NEO spectrometer operating at a 600.13 MHz ^1^H Larmor frequency and equipped with a 5‐mm cryogenic probe Prodigy CTI. All NMR samples were thermally equilibrated at 298.1 K for at least 5 min inside the spectrometer. The following acquisition parameters were used for the CSSF‐TOCSY experiments: 2 × 14 increments; 1.3 s acquisition time; 2 s relaxation delay; and a 70 ms DIPSI‐2 mixing scheme flanked by zero‐quantum filters, for a total acquisition time of 2 min for each selective experiment. ^1^H‐1D, COSY, TOCSY, edited HSQC, and HMBC experiments were carried out. For HSQC, a *J* value of 145 Hz was used; for HMBC, a delay value of 80 ms was used for evolution of long‐range couplings; and for TOCSY, a mixing time of 0.3 s was used. All NMR spectra were processed and analyzed with Bruker Topsin 4.0.6 (Bruker BioSpin GmbH, Rheinstetten, Germany).

### 2.6 Galactinol and Ciceritol Isolation Through HPLC–RID

By using HPLC–RID Method A (injection volume of 10 µL), the galactinol‐rich fraction was manually collected from the outlet port of the RID module, to which a proper capillary was connected for the purpose. In the case of the green coffee sample (geographical origin Ethiopia), the manual fraction collection was repeated for 60 chromatographical runs, and the combined fractions were brought to dryness with a nitrogen stream and then dissolved in 400 µL of water. The galactinol‐rich fraction was finally subjected to NMR analyses. The same procedure was repeated for ciceritol from chickpea, but four chromatographic runs were used.

### 2.7 Ciceritol Isolation by Chickpea Seeds to Be Used as a Standard

Ciceritol isolation procedure was performed according to Bernabe et al. (1993) with some modifications. Dried chickpea seeds (200 g) were finely ground in a batch mill (IKA M20 Universal Mill) and mixed with ethanol–water (8:2 v/v) (500 mL) for 1.5 h at 24°C under stirring. This mixture was filtered through a sintered glass funnel, and the solution was evaporated under vacuum (Rotavapor R114, Buchi). The residue (2.0 g) was mixed with sea sand and a mixture of methanol–ammonia (9:1 v/v) until a fluid mass was obtained. This mass was transferred to the top of a glass column (3 cm i.d.) filled with silica gel G60 (200 g), and the column was eluted with the methanol–ammonia mixture. Fractions (60 mL) were collected, and their content was monitored by thin‐layer chromatography (TLC) according to the literature (Bernabe et al. 1993). Briefly, the TLC analysis was performed as a one‐dimensional chromatography, and it was carried out by eluting on a silica gel plate (Merck TLC Silica gel 60 W F254 S) with a mix of *n*‐butanol/acetone/Milli‐Q water (75:75:25) and a run time of 2 h (Lato et al. 1968). The single fractions were applied to the plate with a capillary tube (1–10 µL) in the usual manner, loading 2 µL. The plate was then placed in a rectangular TLC developing tank containing the solvent mixture. After development, the plate was dried at room temperature and then heated in an oven (Heraus T12) at 110°C until the solvent could no longer be detected (Lato et al. 1968). The separated compounds were visualized by spraying the plate with a freshly prepared solution of 80 mg of naphthoresorcinol in 40 mL of absolute ethanol and 0.8 mL of concentrated sulfuric acid (Bernabe et al. 1993; Hedley 2001). The plate was heated again in the oven at 110°C (10–15 min). After this treatment, well‐defined and vivid spots appear against the white background. The colors developed after spraying were galactose (light blue), sucrose (dark violet), raffinose (violet), and stachyose (light violet) (Waksmunddzka‐Hajnos et al. 2008). Ciceritol may be identified thanks to the galactose monomers, which have a certain intense light blue color. All the fractions collected from the column were analyzed by TLC, and the spots were identified by comparison with standard solutions (1 g/L in Milli‐Q water) of galactose, sucrose, raffinose, and stachyose, and with the aqueous chickpea extract (Bernabe et al. 1993; Waksmunddzka‐Hajnos et al. 2008). HPLC coupled with an RI detector analytical method (Method A) previously described was also used to monitor the most relevant fractions. The fractions containing the spot attributed to ciceritol were pooled together and evaporated under vacuum. The residue was dissolved in water and freeze‐dried. The isolation procedure was performed in duplicate (average yield equal to 11.5 mg). Ciceritol was finally subjected to full NMR characterization.

## 3 Results and Discussion

### 3.1 Investigation of Galactinol as a Marker of Geographical Origin in Green Arabica Coffee

#### 3.1.1 HPLC–RID Method Development and Qualitative Screening of Green *C. arabica* Extracts

**FIGURE 1:**
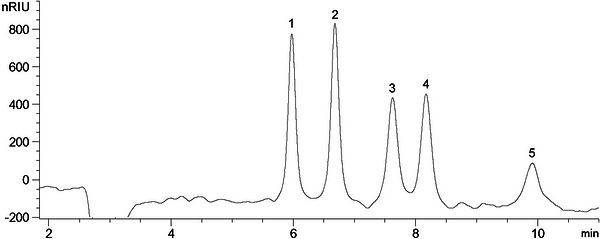
Chromatographic profile (Method A) of a mix of standards: 1: sucrose; 2: *myo*‐inositol; 3: raffinose; 4: galactinol; 5: stachyose.

To establish the chromatographic conditions for the identification and subsequent quantification of galactinol in green coffee beans, a mixture of oligosaccharides and cyclitols standards, including galactinol, was analyzed by HPLC–RID (Method A). The resulting chromatographic profile (Figure 1) showed the following elution order: sucrose, myo‐inositol, raffinose, galactinol, and stachyose, with galactinol eluting at approximately 8 min.

**FIGURE 2:**
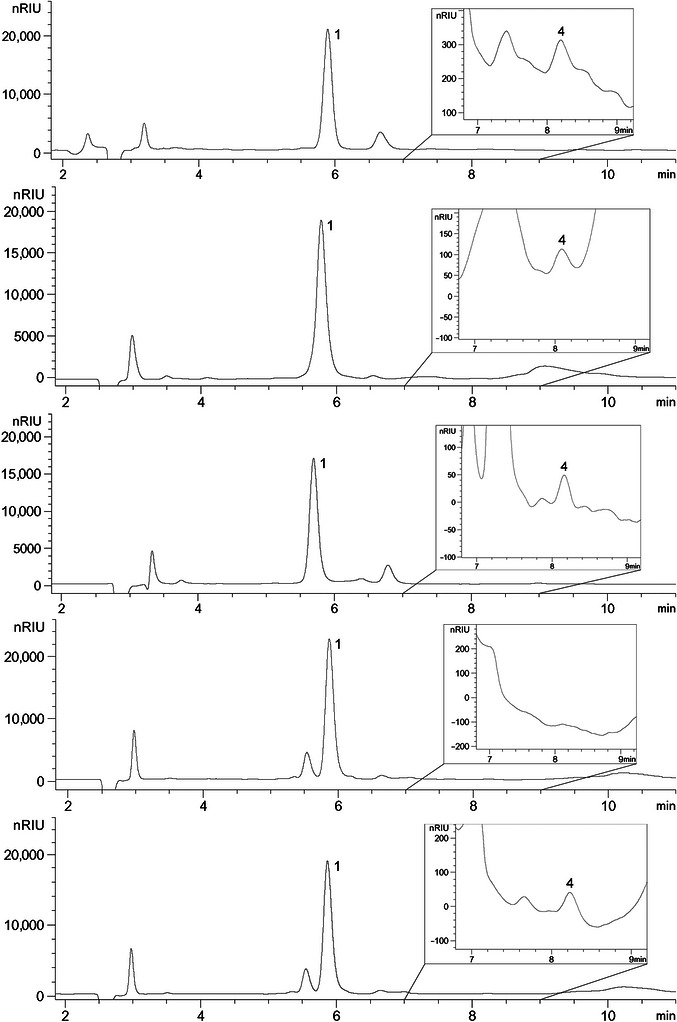
Chromatographic profile (Method A) of green *C. arabica* extracts from several different geographical origins: from the top: Costa Rica, Uganda, Nicaragua, Brazil, and Ethiopia. In the insert, the galactinol peak is shown. 1: sucrose; 4: galactinol.

This method was subsequently applied to the screening of 100 green *C. arabica* extracts from 16 different geographical origins, spanning African, Central American, South American, and Asian regions. A preliminary qualitative analysis of representative chromatographic profiles (Costa Rica, Uganda, Nicaragua, Brazil, and Ethiopia) confirmed sucrose as the dominant oligosaccharide, followed by trace amounts of *myo*‐inositol, raffinose, and stachyose (Figure 2). A peak eluting at around 8 min, consistent with the retention time of galactinol standard, was clearly detected in four of the five samples, being absent only in the Brazilian sample (Figure 2).

#### 3.1.2 Isolation and NMR Structural Confirmation of Galactinol in Green *C. arabica*

**FIGURE 3:**
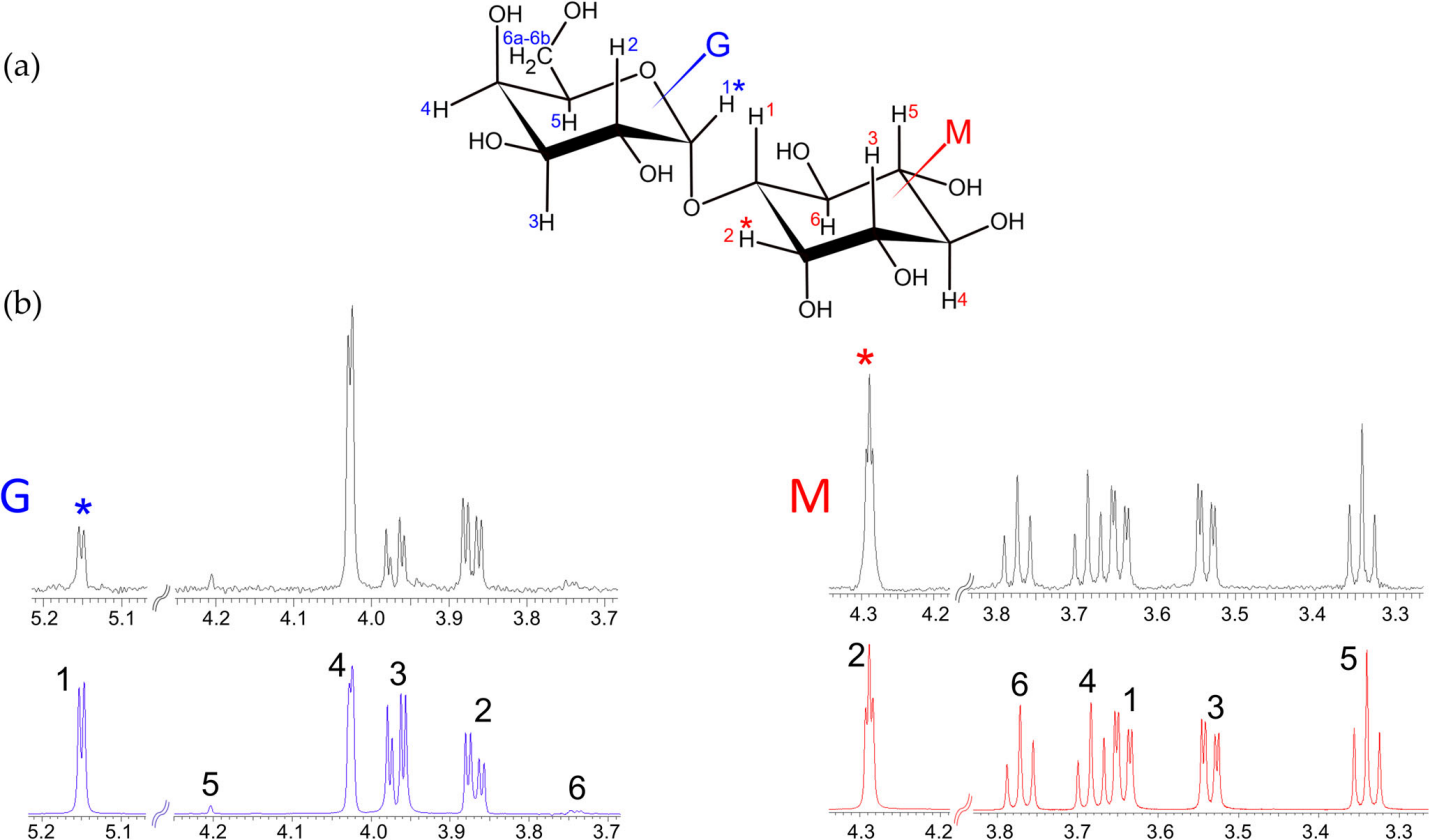
(a) Molecular structure of galactinol (1‐*O*‐α‐d‐galactopyranosyl‐l‐*myo*‐inositol). The α‐d‐galactopyranose ring (G) and the *myo*‐inositol moiety (M) are shown in blue and red, respectively. (b) CSSF‐TOCSY traces recorded for the G ring (left panel) and the M ring (right panel) of galactinol. The black spectra correspond to the galactinol‐rich fraction isolated from Ethiopian green *C. arabica*, whereas the colored spectra refer to the galactinol standard. Resonances for each spin system and colors correspond to the numbering and color legend shown in panel (a).

Prior to quantitative analysis, high‐field NMR spectroscopy was employed to confirm the presence of galactinol (molecular structure in Figure 3a) in green *C. arabica*. A galactinol‐rich fraction was isolated from an Ethiopian sample by collecting multiple chromatographic runs in proximity to the corresponding retention time (i.e., ∼8 min) and subjected to ^1^H‐NMR characterization (Figure 3b). A commercial galactinol standard was analyzed in parallel to guide resonance assignment. The ^1^H NMR spectrum of galactinol standard exhibits severe signal crowding between 3.50 and 4.10 ppm, a region in which nine of the 13 nonexchangeable protons resonate (Figure S1), precluding straightforward assignment of all ^1^H resonances. As is common to all oligosaccharides, the ^1^H NMR spectrum of galactinol can be interpreted as a collection of isolated spin systems, each corresponding to an individual monomeric unit, separated by the glycosidic bonds linking the monomers. To overcome signal overlap and achieve unambiguous resonances assignment of the galactinol moieties, the CSSF‐TOCSY technique was employed (Scettri and Schievano 2022; Schievano et al. 2017; Robinson et al. 2004). Two CSSF‐TOCSY experiments were performed on both the galactinol standard sample (Figures 3b and S1) and the galactinol‐rich fraction extracted by green *C. arabica* (Figure 3b), selectively exciting the anomeric H1 proton of the d‐galactosyl ring (G; Figure 3a) at 5.15 ppm and the most deshielded H2 proton of the polyol moiety (M; Figure 3a) at 4.29 ppm. The resulting CSSF‐TOCSY traces provided isolated signals arising exclusively from each excited sugar ring, thereby enabling unambiguous identification and assignment of the corresponding proton resonances (Table S1). The stereochemistry of the G and M rings was determined in detail by the evaluation of ^3^J ^1^H–^1^H coupling constants derived from the CSSF‐TOCSY traces (Table S1). For the G spin system, a *gauche* conformation was observed for H1–H2, H3–H4, and H4–H5 (^3^J < 5 Hz), while an *anti* conformation was found for H2–H3 (^3^J ∼ 10 Hz). For the *myo*‐inositol ring M, the stereochemistry was assigned: the *gauche* conformation was found for H1–H2 and H2–H3 groups, whereas H3–H4, H4–H5, H5–H6, and H6–H1 protons are in *anti* orientation. These data are in good agreement with previously reported NMR analysis of the galactinol scaffold (Ben Youssef et al. 2016; Gui et al. 2013). The CSSF‐TOCSY signal patterns obtained from the green *C. arabica* extract were indistinguishable from those of the galactinol standard (Figure 3b), unambiguously confirming the presence of this sugar in the analyzed fraction.

#### 3.1.3 Galactinol Occurrence and Quantification Across Geographical Origins

The quantitative HPLC–RID screening of 100 green *C. arabica* samples revealed that galactinol was detected in 31 out of 100 samples, originating from 10 of the 16 geographical origins investigated (Table 1). Although the four macro‐geographical areas considered (Africa, Asia, Central America, and South America) were not uniformly represented in terms of sample number, galactinol was more consistently detected in African and Central American samples, accounting for 52% and 31% of the investigated samples, respectively, whereas it was rarely detected in Asian and South American samples (9% and 11%, respectively) (Table 1). Among all geographical origins, Ethiopia was the only one in which galactinol was consistently detected across all 13 analyzed samples, with a mean content of 86 ± 50 mg/100 g DW, representing the highest average galactinol level observed in this study (Table 1). Quantifiable levels above the LOQ (30 mg/100 g DW) were also found in a fraction of Central American samples: Guatemala (3/8; 63 ± 40 mg/100 g DW), Nicaragua (3/11; 55 ± 27 mg/100 g DW), Costa Rica (4/7; 52 ± 25 mg/100 g DW), and El Salvador (2/3; 39 ± 10 mg/100 g DW). In all these samples, galactinol was identified as a low‐molecular‐weight carbohydrate occurring at relative abundances (approximately 0.04%–0.09% DW) comparable to those of the RFOs (raffinose and stachyose) typically detected in green *C. arabica* beans. In contrast, galactinol was detected at levels at or below the LOQ in samples from Brazil (1/12), Perú (1/1), Honduras (1/8), India (1/6), and Uganda (2/3), and was not detected in samples from Rwanda, Vietnam, Mexico, Colombia, Congo, and the Dominican Republic (Table 1).

**TABLE 1:** Galactinol content in green *Coffea arabica* L. samples from different geographical origins. For origins with quantifiable samples, mean ± SD (mg/100 g DW) is reported. LOQ = 30 mg/100 g DW).

Geographical origin	No. of analyzed samples	Galactinol (mg/100 g DW ± SD)	No. of samples with galactinol	% of samples with galactinol within the same geographical macroarea
Ethiopia	13	86 ± 50	13	52%
Uganda	3	≤30	2	(Africa)
Rwanda	12	<LOD	0	
Congo	1	<LOD	0	
Guatemala	8	63 ± 40	3	31%
Nicaragua	11	55 ± 27	3	(Central America)
Costa Rica	7	52 ± 25	4	
El Salvador	3	39 ± 10	2	
Honduras	8	≤30	1	
Mexico	4	<LOD	0	
Rep. Dominicana	1	<LOD	0	
Brazil	12	≤30	1	11%
Perú	1	≤30	1	(South America)
Colombia	5	<LOD	0	
India	6	≤30	1	9%
Vietnam	5	<LOD	0	(Asia)

Our results indicate that galactinol occurrence is highly variable, both across and within the same geographical region. The absence of galactinol in all 12 Rwandan samples and its sporadic detection at near‐LOQ levels in Ugandan and most Central American, South American, and Asian origins suggest that this compound cannot be considered a universal geographical marker for green *C. arabica*. Its potential diagnostic value appears to be largely confined to Ethiopian coffees, where its consistent and relatively elevated occurrence may support its use as a complementary indicator within a broader multimarker analytical framework. The high intra‐geographical origin variability suggests that galactinol levels may be influenced by additional factors beyond geographical origin, such as genetic diversity, environmental conditions, or postharvest processing practices. In this regard, a recent study reported that galactinol synthase (GolS), the enzyme responsible for galactinol biosynthesis in higher plants, including coffee, is upregulated under abiotic stress conditions such as drought and temperature fluctuations, potentially resulting in increased galactinol accumulation (dos Santos and Vieira 2020). Although a direct link between these factors and the variability observed in the present study cannot be established, this regulatory mechanism may at least partially account for the heterogeneous galactinol distribution observed across and within geographical origins.

### 3.2 Evaluation of Galactinol Specificity in Coffee Authenticity Assessment

The suitability of galactinol as a traceability and authenticity marker for green *C. arabica* depends not only on its occurrence across geographical origins but also on its specificity with respect to matrices commonly involved in coffee adulteration. Among these, legume seeds and particularly chickpeas (*Cicer arietinum* L.) are well‐documented coffee adulterants (Sezer et al. 2018). The variable occurrence of galactinol in *C. arabica*, combined with its possible presence in adulterant matrices, may therefore represent a critical limitation for authenticity and traceability applications. To address this issue, the presence of galactinol was investigated in a chickpea aqueous extract by subjecting it to the same HPLC–RID and NMR analyses employed for green coffee samples. In this legume, the most abundant galactosyl cyclitol reported to date is the trisaccharide ciceritol (*O*‐α‐d‐galactopyranosyl‐(1→6)‐*O*‐α‐d‐galactopyranosyl‐(1→2)‐1d‐4‐*O*‐methyl‐*chiro*‐inositol) (Berrios et al. 2010; Sánchez‐Mata et al. 1998; Quemener and Brillouet 1983). Since ciceritol is absent in coffee beans, it may represent a useful marker of adulteration. By contrast, the occurrence of galactinol in chickpeas remains controversial, as its presence has not been consistently reported across different studies (Quemener and Brillouet 1983; Xiaoli et al. 2008).

#### 3.2.1 NMR Analysis Put in Evidence an Unexpected Co‐Elution of Galactinol With the Adulterant Ciceritol

**FIGURE 4:**
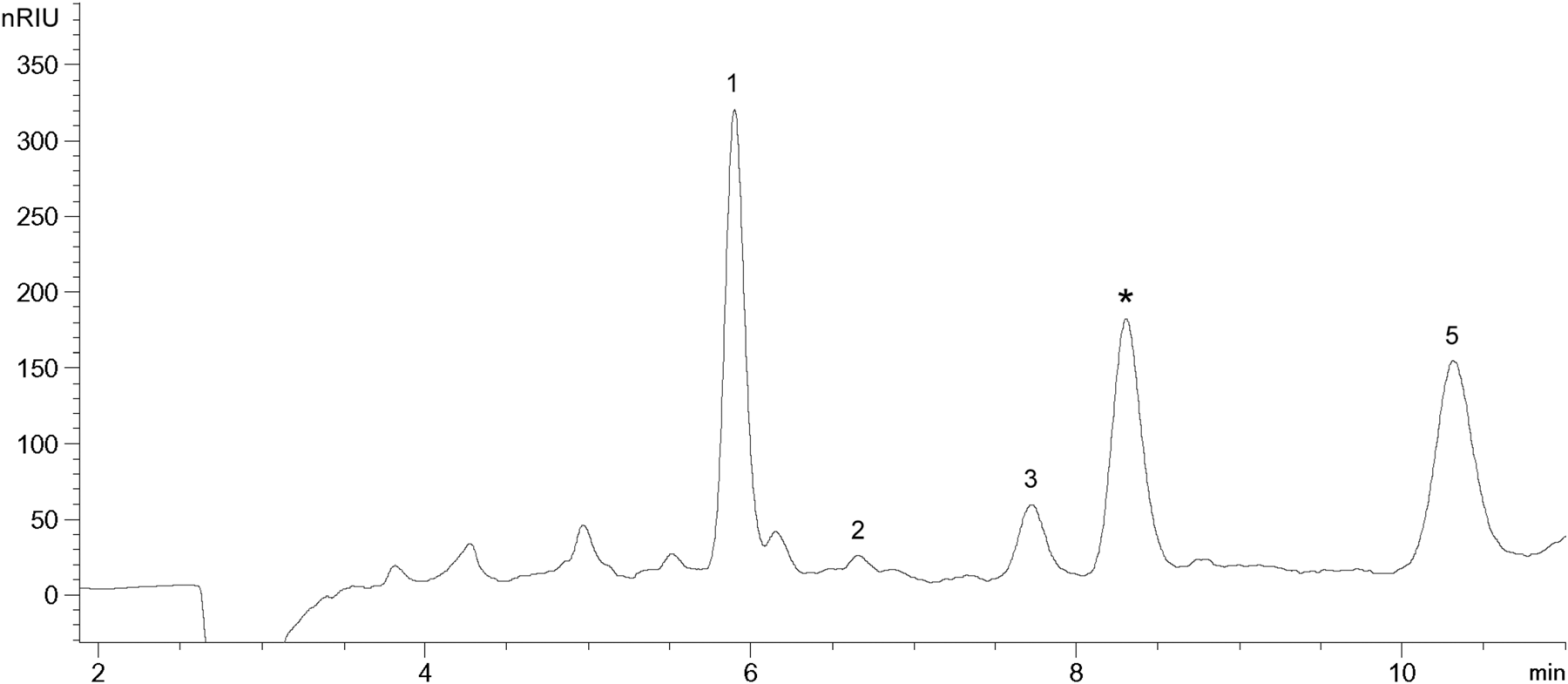
Chromatographic profile (Method A) of an aqueous extract of chickpeas: 1: sucrose; 2: *myo*‐inositol; 3: raffinose; *: tentative galactinol; 5: stachyose.

**FIGURE 5:**
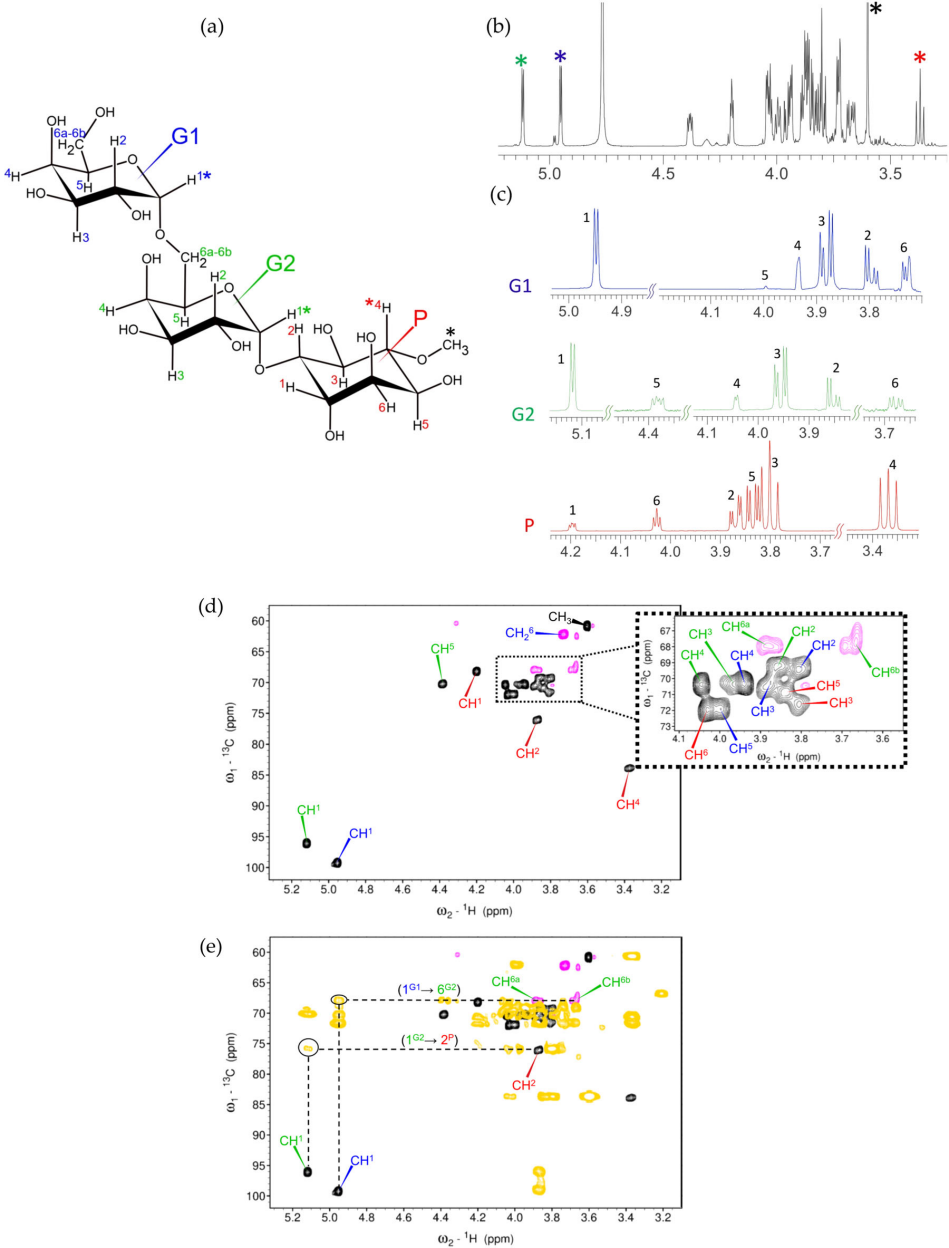
(a) Molecular structure of ciceritol (*O*‐α‐d‐galactopyranosyl‐(1→6)‐*O*‐α‐d‐galactopyranosyl‐(1→2)‐1d‐4‐*O*‐methyl‐*chiro*‐inositol). The two α‐d‐galactopyranose rings (named G1 and G2) are colored in blue and green, respectively, whereas the D‐*chiro* inositol ring (i.e., the pinitol moiety, named P) is colored in red. (b) 1D ^1^H spectrum acquired on the ciceritol standard isolated from chickpea extract. The three groups of resonances, marked by green, blue, and red asterisks, correspond, respectively, to the anomeric proton (H1) of the G2 and G1 rings, and the H4 proton of the P ring. The resonance of the methyl group of the pinitol ring is indicated with a black asterisk. (c) CSSF‐TOCSY spectra recorded for G1, G2, and P rings of ciceritol isolated from chickpea. The signals of each spin system are assigned based on the numbering and color legend of panel (a). (d) ^1^H–^13^C edited HSQC spectrum acquired for ciceritol, with all the 19 ^13^C resonances fully assigned. The expanded plot shows the cross‐peaks assignment in a region of the spectrum particularly crowded. (e) ^1^H–^13^C HMBC spectrum (yellow) overlaid with the ^1^H–^13^C edited HSQC spectrum of ciceritol reveals the long‐range connectivity between the anomeric H1 of ring G1 and C6 of ring G2 and between the anomeric H1 of ring G2 and C2 of ring P.

**FIGURE 6:**
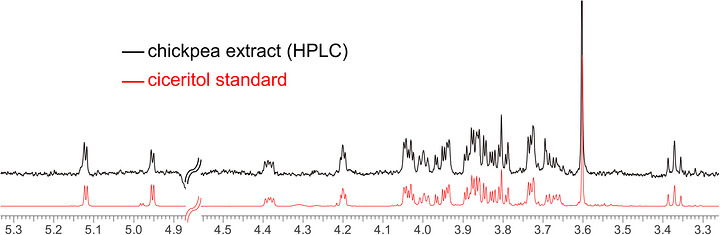
Comparison between the 1D ^1^H NMR spectra of the chromatographic peak marked with an asterisk (*) in Figure 4 (black spectrum) and that of the isolated ciceritol standard (red spectrum).

In Figure 4, the chromatogram obtained by subjecting an aqueous extract of chickpea seeds to the same HPLC–RID analysis used for green coffee (Method A) is reported. In addition to sucrose, *myo*‐inositol, raffinose, and stachyose, the chromatogram clearly shows a peak (marked with an asterisk in Figure 4) with a retention time of about 8 min, coincident with that of the galactinol standard (Figure 1). The fraction eluting at the corresponding retention time was collected and subjected to ESI–MS analyses. The resulting mass spectrum exhibited a signal corresponding to a mass of 518.2 Da (Figure S2), which is not consistent with the expected molecular weight of galactinol (182.2 Da) but rather that of ciceritol (518.5 Da), whose molecular structure is shown in Figure 5a. To unambiguously confirm the identity of the co‐eluting compound, NMR analysis was performed on the chromatographic fraction isolated from chickpeas, following the same approach employed for the galactinol‐rich fraction from green *C. arabica*. In the absence of a commercially available analytical standard for ciceritol, the compound was isolated from dried chickpea seeds using a dedicated procedure, and the resulting material was then used as an in‐house reference standard. The ^1^H‐NMR spectrum of the standard (Figure 5b) readily revealed the signals expected for the two anomeric protons of the d‐galactosyl rings of ciceritol (G1 and G2; Figure 5a), which resonate at 4.95 and 5.12 ppm, respectively (Table S2). However, severe signal overlap in the remaining proton resonances required the CSSF‐TOCSY technique for complete and unambiguous assignment. Three CSSF‐TOCSY experiments were acquired (Figure 5c), selectively exciting the anomeric protons of the G1 and G2 rings of ciceritol and the H4 proton at 3.37 ppm of the polyol moiety (i.e., pinitol) (P ring; Figure 5a), enabling the full assignment of all proton resonances of ciceritol (Table S2), in agreement with a previously published NMR assignment of ciceritol from lentils (Bernabe et al. 1993). The analysis of ^3^J ^1^H–^1^H coupling constants derived from the CSSF‐TOCSY traces (Table S2) allowed us to confirm the stereochemistry expected for each of the three rings of ciceritol. Complete structural elucidation was achieved through two‐dimensional ^1^H–^13^C correlation NMR experiments. The ^13^C resonances of the sugar were initially assigned using an edited HSQC pulse sequence (Heteronuclear Single Quantum Coherence) (Figure 5d), while inter‐residue connectivities were established through the HMBC (Heteronuclear Multiple Bond Correlation) correlation (Figure 5e). Specifically, the HMBC correlations between the anomeric H1 of ring G1 and C6 of ring G2 (4.95 and 67.9 ppm, respectively) and between the anomeric H1 of ring G2 and C2 of ring P (5.12 and 76.1 ppm, respectively) were unambiguously detected, as shown in Figure 5e. Additionally, the ^13^C resonances of the anomeric protons (99.1 and 96.1 ppm for G1 and G2, respectively) indicated the α configuration (Bernabe et al. 1993). Taken together, these data confirm the α‐(1→6) linkage between G1 and G2 and the α‐(1→2) linkage between G2 and the D‐pinitol ring (P), consistent with the established structure of ciceritol. The 1D ^1^H NMR spectrum of the fraction isolated from chickpea extract showed signal patterns indistinguishable from those of ciceritol standard (Figure 6), while no signals attributable to galactinol were detected. These results unambiguously demonstrate that galactinol is absent in the analyzed chickpea extract and that the chromatographic peak observed at about 8 min under Method A is exclusively attributable to ciceritol.

While these findings preliminarily support the specificity of galactinol as a traceability and authenticity marker for green coffee, they also reveal an unexpected and critical analytical interference between galactinol and ciceritol under the chromatographic conditions of Method A, with direct implications for coffee authenticity assessment

#### 3.2.2 Chromatographic Resolution of Galactinol and Ciceritol Co‐Elution

The observed co‐elution between galactinol and ciceritol poses a problem from the standpoint of coffee authenticity, and therefore, we wanted to explore an HPLC–RID method under completely different conditions than those described so far. Specifically, by varying both the type of chromatographic column and the mobile phase and simultaneously raising the column and detector temperatures (Method B), it was possible to obtain separation conditions in which the galactinol peak was completely separated from the other oligosaccharides and cyclitols used as standards. In Table 2, retention times of the different standards determined by using both Method A and Method B are reported. As shown in Table 2, the co‐elution of galactinol and ciceritol observed under Method A conditions is fully resolved by Method B, with the two compounds exhibiting retention times of 16.36 and 13.53 min, respectively. However, Method B introduces a different analytical limitation, namely, the co‐elution of raffinose and ciceritol (retention times of 13.31 and 13.53 min, respectively), which should be considered when applying this method to complex matrices.

**TABLE 2:** Retention time of the different standards by adopting Method A and Method B.

Compound	Retention time (min) Method A	Retention time (min) Method B
Sucrose (1)	5.97	14.42
*Myo*‐inositol (2)	6.68	20.83
Raffinose (3)	7.62	13.31
Ciceritol (*)	8.36	13.53
Galactinol (4)	8.17	16.36
Stachyose (5)	10.00	12.63

## 4 Conclusion

Despite the relevance of galactinol in green *C. arabica* beans, particularly regarding its potential use as a marker of geographical origin, quantitative reports on its occurrence in green coffee seeds remain very scarce. In this study, galactinol was isolated for the first time from green *C. arabica* L. beans and structurally identified by high‑field NMR spectroscopy. Quantitative HPLC–RID screening of 100 green Arabica coffee samples from 16 geographical origins revealed a highly variable and not ubiquitous occurrence of galactinol, with a higher incidence in samples from Africa and Central America. Overall, galactinol was detected in 31% of the analyzed samples, with a particularly consistent presence in Ethiopian coffees, in contrast to samples from several other origins where it was detected sporadically or not at all. Although these results suggest that galactinol may represent a potential marker for specific coffee‑growing regions, particularly Ethiopia, its heterogeneous distribution observed among samples from other geographical origins, including those belonging to the same macro‐geographical area, indicates that, at present, galactinol cannot be considered a universal geographical marker for green *C. arabica*. Its potential value may instead lie in its use as a complementary indicator within a broader multimarker analytical framework. Our work paves the way for further analysis on larger sample datasets to more deeply understand the link between the high variability observed here in galactinol levels among green coffee samples and their geographical origin.

A critical analytical issue highlighted in this work is the co‑elution of galactinol with ciceritol under conventional HPLC‑RID conditions commonly employed for sugar profiling (Method A). Through a combination of ESI–MS analysis and comprehensive NMR characterization, the chromatographic signal previously attributable to galactinol in chickpea extracts was unambiguously assigned to ciceritol, while no galactinol was detected in the analyzed chickpea extract. The development of an alternative HPLC‑RID method (Method B) allowed the complete resolution of galactinol from potential interferents, thereby improving the selectivity and reliability of its determination in green coffee. The two methods should therefore be regarded as complementary: Method A for routine qualitative and quantitative screening of green coffee samples and Method B for confirmatory analysis in authenticity assessment contexts, where the selective determination of galactinol in the presence of potential adulterant‐derived interferents is required. These results underline the importance of integrating chromatographic techniques with structure‑specific spectroscopic methods, such as NMR, to identify weaknesses in routine analytical methods and to avoid misinterpretation arising from co‑eluting compounds.

In conclusion, beyond advancing the knowledge of galactinol occurrence in green *C. arabica*, this work provides a robust framework for authenticity studies and highlights the need for highly selective methods when minor carbohydrates are considered as potential markers of geographical origin or product adulteration.

## Author Contributions

**Elisabetta De Angelis**: methodology, data curation. **Simone Fabbian**: conceptualization, methodology, data curation, investigation, formal analysis, writing – original draft, writing – review and editing. **Elisabetta Schievano**: conceptualization, methodology, data curation, formal analysis, writing – original draft, writing – review and editing, investigation. **Elena Guercia**: data curation, methodology. **Arianna Fornasari**: methodology, data curation. **Elena Piva**: methodology, data curation. **Michele Pozzebon**: methodology, data curation. **Luciano Navarini**: conceptualization, methodology, formal analysis, writing – original draft, writing – review and editing.

## Conflicts of Interest

The authors declare no conflicts of interest.

## Supporting information




**Supplementary Material**: jfds71238‐sup‐0001‐SuppMat.docx
