# Supplementary material for: NMR Identification and Quantification of Galactinol in Green Coffea arabica: Implications for Traceability and Adulteration
Source: J Food Sci. 2026 Jun 24;91(6):e71238. doi: 10.1111/1750-3841.71238 (PMC13292189; doi:10.1111/1750-3841.71238)
Supplement: Supplementary file 1 — Supplementary Material: jfds71238‐sup‐0001‐SuppMat.docx [file JFDS-91-0-s001.docx]

**NMR Identification and Quantification of Galactinol in Green *Coffea* *Arabica*:**

**implications for Traceability and Adulteration**

Elisabetta De Angelis^1a^, Simone Fabbian^2a,#^, Elisabetta Schievano^2#^, Elena Guercia^1^, Arianna Fornasari^3^, Elena Piva^4^, Michele Pozzebon^4^, and Luciano Navarini^5^

1 Aromalab illycaffè spa, Area Science Park, Padriciano 99, 34149 Trieste, Italy

2 Department of Chemical Sciences, University of Padova, via Marzolo 1, 35131 Padova, Italy

3 Department of Medical and Surgical Sciences, University of Bologna, via Irnerio 49, 40126 Bologna, Italy

4 dtoLABS, via Pozzuoli 13C/13D, 30038 Spinea (VE), Italy

5 illycaffè spa, via Flavia 110, 34147 Trieste, Italy

^a^ contributed equally

# Corresponding author: Simone Fabbian ([simone.fabbian@unipd.it](mailto:simone.fabbian@unipd.it)) Elisabetta Schievano (elisabetta.schievano@unipd.it)

| 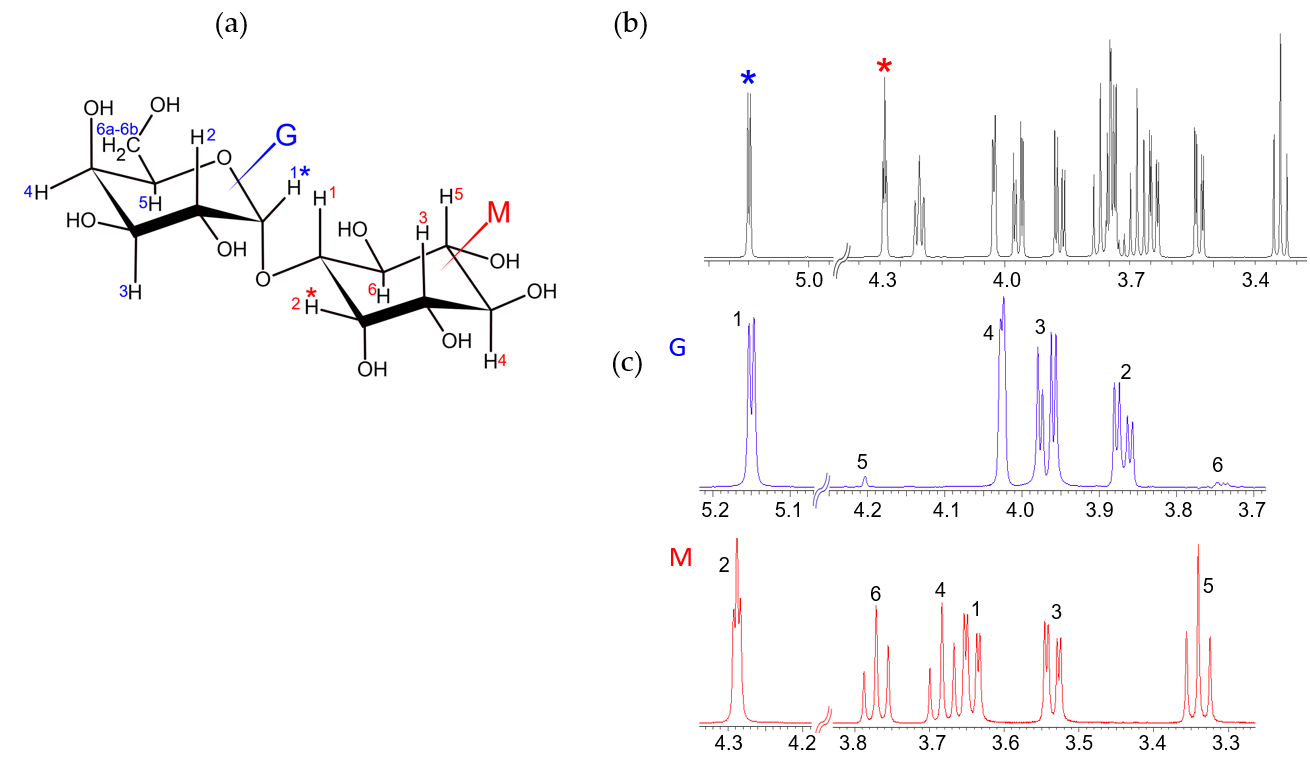 |
| --- |
| **Figure S1**. (**a**) Molecular structure of galactinol (1-O-α-D-galactopyranosyl-L-*myo*-inositol). The α-D-galactopyranose ring (G) is shown in blue, whereas the *myo*-inositol moiety (M) is coloured in red. (**b**) 1D ^1^H NMR spectrum acquired on the galactinol standard sample. The two groups of resonances, marked by blue and red asterisks, correspond respectively to the H1 and H2 protons of the G and M rings (**c**) CSSF-TOCSY spectra recorded for G and M rings of galactinol standard. The resonances for each spin system are assigned based on the numbering and colour legend presented in panel **a**. |

| 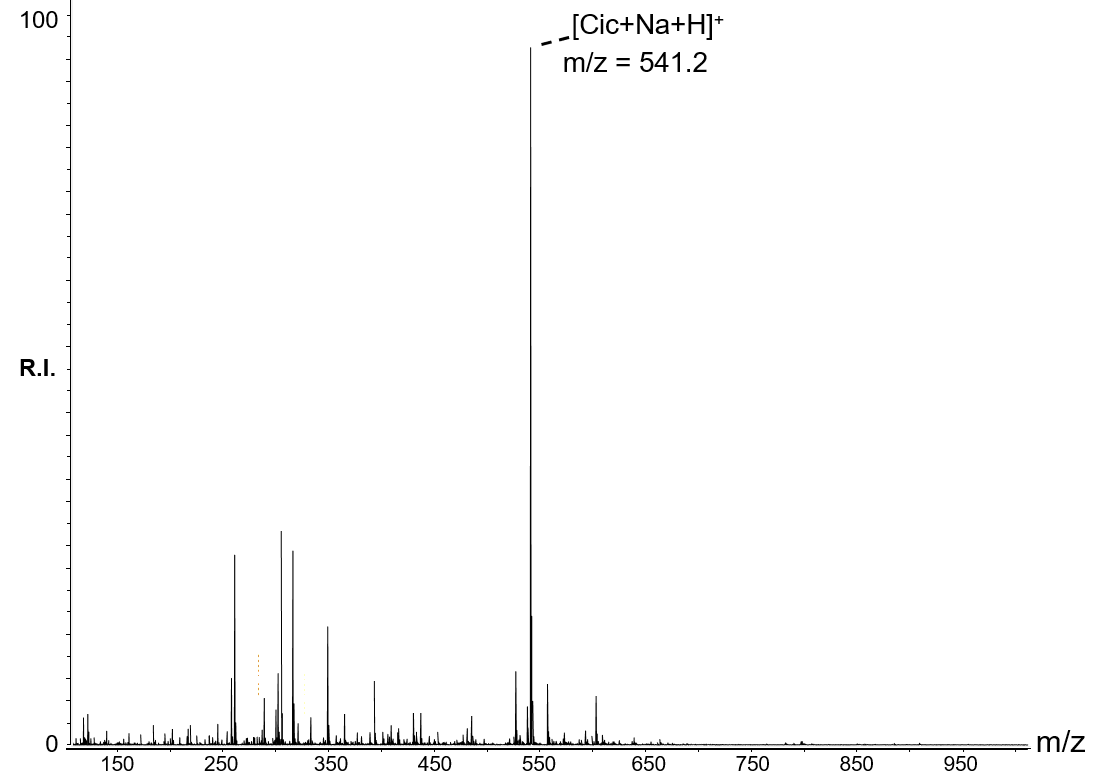 |
| --- |
| **Figure S2**. ESI-MS spectrum of ciceritol (Cic) in milliQ water:acetonitrile 50:50 with 0.1% (v/v) formic acid. |

**Table S1**

^1^H and ^13^C NMR chemical shifts and ^1^H coupling constants of galactinol in D_2_O at 25°C.

| **Position** | **Chemical shift**  **(ppm)** | **Coupling constant**  **(Hz)^a^** | |
| --- | --- | --- | --- |
|  | δ_1H_ | *^n^J_H-H_* | |
| **Galactopyranosyl (ring G)** |  | | |
| 1 | 5.15 (d) | ^3^*J*_1,2_ | 3.9 |
| 2 | 3.87 (dd) | ^3^*J*_2,3_ | 10.3 |
| 3 | 3.97 (dd) | ^3^*J*_3,4_ | 3.3 |
| 4 | 4.03 (dd) | ^3^*J*_4,5_ | <1.0 |
| 5 | 4.20 (ddd) (m) | ^3^*J*_5,6a_;^3^*J*_5,6b_ | 6.6 |
| 6a | 3.74 (m) | ^2^*J*_6a,6b_ | n.d. |
| 6b |  |  |  |
| ***Myo*-inositol**  **(ring M)** |  | | |
| 1 | 3.64 (dd) | ^3^*J*_1,2_ | 2.6 |
| 2 | 4.29 (dd) (m) | ^3^*J*_2,3_ | 2.8 |
| 3 | 3.53 (dd) | ^3^*J*_3,4_ | 10 |
| 4 | 3.68 (dd) (m) | ^3^*J*_4,5_ | >8.0 |
| 5 | 3.34 (dd) (m) | ^3^*J*_5,6_ | >8.0 |
| 6 | 3.77 (dd) (m) | ^3^*J*_1,6_ | 10 |

^a^ value determined from CSSF-TOCSY experiments.

**Table S2**

^1^H and ^13^C NMR chemical shifts and ^1^H coupling constants of ciceritol in D_2_O at 25°C.

| **Position** | **Chemical**  **shift**  **(ppm)** | | **Coupling**  **constant**  **(Hz)^a^** | |
| --- | --- | --- | --- | --- |
|  | δ_1H_ | δ_13C_ | *^n^J_H-H_* | |
| **Galactopyranosyl (ring G1)** |  | | | |
| 1 | 4.95 (d) | 99.1 | ^3^*J*_1,2_ | 3.9 |
| 2 | 3.80 (dd) | 69.4 | ^3^*J*_2,3_ | 10.0 |
| 3 | 3.88 (dd) | 70.2 | ^3^*J*_3,4_ | 3.6 |
| 4 | 3.94 (dd) | 70.3 | ^3^*J*_4,5_ | <2.0 |
| 5 | 3.99 (m) | 71.9 | ^3^*J*_5,6_ | n.d. |
| 6a | 3.73 (m) | 62.1 | ^2^*J*_6a,6b_ | n.d. |
| 6b |  |  |  |  |
| **Galactopyranosyl (ring G2)** |  | | | |
| 1 | 5.12 (d) | 96.1 | ^3^*J*_1,2_ | 3.9 |
| 2 | 3.85 (dd) | 69.2 | ^3^*J*_2,3_ | 10.0 |
| 3 | 3.96 (dd) | 70.2 | ^3^*J*_3,4_ | 3.4 |
| 4 | 4.04 (dd) | 70.3 | ^3^*J*_4,5_ | <3.0 |
| 5 | 4.38 (ddd) | 70.2 | ^3^*J*_5,6a_;^3^*J*_5,6b_ | 4.3; 8.2 |
| 6a | 3.67 (m) | 67.9 | ^2^*J*_6a,6b_ | 10.4 |
| 6b | 3.87 (m) |  |  |  |
| **Pinitol**  **(ring P)** |  | | | |
| 1 | 4.20 | 68.2 | ^3^*J*_1,2_ | 3.0 |
| 2 | 3.87 | 76.1 | ^3^*J*_2,3_ | 10.9 |
| 3 | 3.80 | 71.6 | ^3^*J*_3,4_ | >8.0 |
| 4 | 3.37 | 83.9 | ^3^*J*_4,5_ | 10.1 |
| 5 | 3.84 | 70.8 | ^3^*J*_5,6_ | 3.4 |
| 6 | 4.03 | 71.9 | ^3^*J*_1,6_ | 4.3 |
| CH_3_ | 3.60 | 60.8 |  | |

^a^ value determined from CSSF-TOCSY experiments.
